# Dietary resistant starch preserved through mild extrusion of grain alters fecal microbiome metabolism of dietary macronutrients while increasing immunoglobulin A in the cat

**DOI:** 10.1371/journal.pone.0241037

**Published:** 2020-11-03

**Authors:** Matthew I. Jackson, Christopher Waldy, Dennis E. Jewell

**Affiliations:** Pet Nutrition Center, Hill's Pet Nutrition, Inc., Topeka, KS, United States of America; University of Illinois, UNITED STATES

## Abstract

Dietary digestion-resistant starch (RS) provides health benefits to the host via gut microbiome-mediated metabolism. The degree to which cats manifest beneficial changes in response to RS intake was examined. Healthy cats (N = 36) were fed identically formulated foods processed under high (n = 17) or low (n = 19) shear extrusion conditions (low and high RS levels [LRS and HRS], respectively). Fecal samples collected after 3 and 6 weeks' feeding were assayed for stool firmness score, short-chain fatty acids, ammonia, and changes to the global metabolome and microbiome; fecal immunoglobulin A (IgA) was analyzed at week 6. Few differences were seen in proximate analyses of the foods; stool firmness scores did not differ. In cats consuming HRS food, concentrations of fecal butyrate and the straight chain:branched chain fatty acid ratio were significantly greater in feces at both weeks 3 and 6, while fecal ammonia was reduced at week 6 relative to feces from LRS-fed cats. Fecal IgA concentrations were significantly higher at week 6 with HRS food. RS consumption altered 47% of the fecal metabolome; RS-derived sugars and metabolites associated with greater gut health, including indoles and polyamines, increased in the cats consuming HRS food relative to those fed the LS food, while endocannabinoid N-acylethanolamines decreased. Consumption of HRS food increased concentrations of the ketone body 3-hydroxybutyrate in feces and elevated concentrations of reduced members of NADH-coupled redox congeners and NADH precursors. At the microbiome genus-level, 21% of operational taxonomic units were significantly different between food types; many involved taxa with known saccharolytic or proteolytic proclivities. Microbiome taxa richness and Shannon and Simpson alpha diversity were significantly higher in the HRS group at both weeks. These data show that feline consumption of grain-derived RS produces potentially beneficial shifts in microbiota-mediated metabolism and increases IgA production.

## Introduction

The lower gut of monogastric animals harbors communities of microbiota (ie, the microbiome). In this mutualistic relationship, dietary nutrients escaping host digestion and absorption become available to the microbiome while the microbiome concomitantly generates a number of small molecule metabolites (postbiotics) that act locally in the gastrointestinal tract and are also reabsorbed across the colon to systemic circulation [1]. Microbiome function and production of postbiotics have a large impact on host health, so characterization of these metabolites can provide insights into optimal health [2, 3]. Food processing through extrusion can increase the digestibility and bioavailability of nutrients to the host while restricting their access to the colonic microbiota. During extrusion, the ingredients are cooked using both mechanical shear force and heat; each of these parameters can be scaled on a spectrum from mild to intense. Depending on conditions, extrusion can improve the digestibility of protein [4] and amino acids [5] and increase fiber-carbohydrate solubility by degrading plant cell walls [6]. Most foods destined for consumption by companion animals are extruded and the consequences for pet nutrition have been reviewed [7]. Grains are prominent sources of dietary starch and serve as a source of energy, fiber, and protein for companion animals and also enable formation of a dry, extruded pet food kibble with appropriate expansion.

Nutrient digestibility is typically paramount in food production, and extrusion aids in achieving this goal. Recently however, mild conditions of grain extrusion with reduced mechanical shear force and heat have been utilized to retain type 2 ungelatinized dietary digestion-resistant starch (RS) [8]. Additionally, decreased shear force reduces scission of high molecular weight starch [9]; both RS and high molecular weight starch polymers are less digestible and thus arrive relatively intact to the large intestine.

Increasing RS through mild food processing can provide health benefits via the intermediacy of the action of gut microbes to ferment RS to beneficial catabolic products, as has been observed for humans [10] and dogs [11]. Varying the conditions of grain extrusion has been shown to modify microbiome composition *in vitro* as well as the degree to which microbiota produce fermentation products [12]. Domesticated felines (*Felis catus*) are obligate carnivores; however, RS consumption might be expected to influence their microbiome composition and activity since phylogeny and functional gene content of the domesticated cat microbiome is similar to that of omnivores [13] and also markedly different from that of both captive and non-captive wild felines [14]. Although there are reports examining the effects of non-starch polysaccharide fibers added to diets of cats [15–18], there are no published reports of the microbiome effects of dietary intervention in cats with foods designed to vary in RS concentrations.

The metabolic functions of the microbiome that act on dietary constituents can roughly be classified as saccharolytic, proteolytic, and lipolytic. The microbiome can carry out the saccharolysis of intact starch granules in the colon to produce fermentative postbiotics including short-chain fatty acids (SCFA) [19], which have a number of beneficial physiological effects [20]. Dietary protein escaping digestion can be subjected to proteolysis by the microbiome and the resultant amino acids putrefied to products, including branched-chain fatty acids (BCFA) [21], which may be associated with disease [22] or beneficial impacts on host physiology [23]. Lipolysis of undigested dietary fat can lead to production of postbiotics with health-promoting effects to improve gut barrier integrity and reduce inflammatory conditions [24, 25], and commensal microbes generate bioactive lipids *de novo* that impact host physiology [26].

In this study, the physiological effect of consumption of identically formulated food processed under extrusion conditions generating either lower or higher concentrations of RS on microbiome saccharolysis, proteolysis, and lipolysis was examined in cats.

## Materials and methods

### Food production

Identically formulated foods (Table 1) that met the maintenance nutrition requirements outlined by the Association of American Feed Control Officials (AAFCO) and National Research Council were extruded with either high or low shear force. Extrusion was performed with a Wenger X-115 extruder (Wenger model 7 preconditioner; 115 mm outside diameter screw element) at the highest or lowest maximal shear rates that would provide a finished kibble with appropriate aesthetic properties. The higher specific mechanical energy in the high shear extrusion was due to high shaft rpm, low mass throughput, and reduced die open area (that increased the shear rate; S1 Fig). After extrusion, the formulations were dried and coated with palatants and fat.

**Table 1:** Study food formulation, proximate analysis, and digestibility.

	LRS	HRS
Ingredient, % dry matter	Formulation	
Poultry by-product meal (low ash)	37.3	
Brewer's rice	18.5	
Corn gluten meal	11.8	
Whole grain corn	10.3	
Pork fat	9.9	
Cellulose	2.8	
Palatant (spray-dried chicken)	2.0	
Lactic acid	1.2	
Palatant (liver digest)	1.2	
Potassium chloride	1.1	
Choline chloride	0.9	
Calcium sulfate	0.8	
Beet pulp	0.7	
Methionine, dL	0.5	
Vitamin E oil	0.3	
Palatant (dried yeast)	0.2	
Sodium chloride, iodized	0.2	
Vitamin mix	0.2	
Mineral mix	0.1	
Taurine	0.1	
Phosphoric acid	< 0.1	
**Proximate analysis, %**		
Moisture	4.9	5.4
Fat	18.9	19.0
Protein	37.6	38.0
Neutral detergent fiber	9.7	8.4
Ash	6.1	6.1
Crude fiber	3.2	3.1
Non-gelatinized starch (putative RS)	0.5	7.9
**Digestibility**		
Apparent dry matter digestibility, %	85.2	82.5
Apparent protein digestibility, %	86.8	83.0
Apparent fat digestibility, %	94.0	93.2
Apparent fiber digestibility, %	22.6	20.1
Apparent NFE digestibility, %	91.0	89.0
Diet metabolic energy-AAFCO tested, kcal/kg	4371	4286

AAFCO, Association of American Feed Control Officials; HRS, high resistant starch; LRS, low resistant starch; NFE, nitrogen-free extract.

### Food and digestibility analyses

Before the trial described here, each food was initially screened for digestibility in separate digestibility feeding studies following the AAFCO quantitative collection protocol [27]. In these preliminary studies, animals were fed to maintain body weight, and water was freely available at all times. Each test included 6 adult cats and consisted of two phases: a pre-collection acclimation period of 9 days followed by a second phase lasting 5 days with total collection of feces. Analytical measurements for food-related energy, moisture, protein, fat, fiber, and ash and the corresponding nutrients in feces (fecal undigested nutrients) were performed as outlined by AAFCO. Crude fiber was measured according to the AOAC method 962.09. Digestibility coefficients for dry matter, nitrogen-free extract (NFE; putative digestible carbohydrate), and fiber were calculated as apparent digestibility [(consumed—fecal)/consumed]. Analytical measurements for food-related energy, moisture, protein, fat, fiber, and ash and the corresponding nutrients in feces were performed as outlined by AAFCO. Total dietary starch was approximated as putative digestible carbohydrate and is calculated from digestibility data as NFE, where NFE = 100%–(crude fiber % + crude protein % + crude fat % + ash % + moisture %). Percent cook quantitatively determines the degree of gelatinized starch in cereal-based foodstuffs based on the principle that gelatinized starch is readily digested by glucoamylase. Total starch is determined similarly with the exception that intact or raw starch in the sample is gelatinized prior to enzymatic digestion. Percent cook was determined on a YSI 2700 SELECT Biochemistry Analyzer (YSI Inc., Yellow Springs, Ohio, USA), and was calculated as (% gelatinized starch in food/% total starch in food) x 100. Proximate analysis was carried out as previously described [28]. For rapid visco analysis (RVA), samples were frozen at -70°C, ground, and passed through a US#40 sieve [29]. Measured moisture was used to calculate sample size and water addition. Samples were run on a 23-minute profile and analyzed as previously described [30].

### Animals and experimental design

Cats were individually housed in environments promoting social interaction, had access to natural light that varied with season, had daily opportunity to exercise and interact with caretakers, and were fed to maintain healthy body weight. The study protocol was reviewed and approved by the Institutional Animal Care and Use Committee, Hill's Pet Nutrition, Topeka, KS, USA (Protocol Number: FP644a.1.2.0-A-F-D-ADH-MULTI-91-GI), and complied with the National Institutes of Health guide for the care and use of laboratory animals as well as those from the US National Research Council and US Public Health Service [31]. All cats were domestic shorthair breed and were spayed or neutered. Cats were owned by the funders of this research, who gave permission for them to be included in this study. At the conclusion of the study, all cats were healthy and returned to the colony. Inclusion criteria was for healthy cats, considered as no evidence of chronic systemic disease in physical examination, complete blood count, serum biochemical analyses, urinalysis, or fecal examination for parasites. All cats completed the trial with no adverse events. Fresh food was offered daily with amounts calculated to maintain body weight, with electronic feeders that recorded daily food intake (g/day) for each cat; water was available ad libitum.

Cats were randomized by age, weight, and sex into two groups, where they received the high shear (low RS [LRS]) food or the low shear (high RS [HRS]) food (S1 Table). Caretakers were blinded to the food received, and each cat received only one of the foods. Body weight was assessed at baseline and every week for the duration of the study.

The study was a randomized, 7-week, longitudinal feeding trial with fecal collections carried out under IACUC-approved protocols. As felines do not reliably produce stools on a daily basis, two replicate fecal collections for each cat were attempted within a four-day period initiated at the outset of weeks 4 and 7. Analysis of IgA was carried out only on the first successful collection at week 7, and metabolomics analysis was only carried out on the first successful collection at both weeks 4 and 7. As initiation of stool collection occurred on the first day of weeks 4 and 7, there were 3 and 6 full weeks of feeding the experimental foods prior to sample collection periods. Hereafter, we refer to these collection timepoints as weeks 3 and 6. All cats were healthy following the study.

### Fecal analysis

Stool production from cats is not regular or predictable. Therefore, in order to collect fresh feces, between the hours of 07:00 to 15:00 on collection days, caretakers visited the litter boxes every 15 minutes to assess production of stool. When a stool was observed, it was immediately scored on a 5-point scale on which a higher score indicates firmer stool [32]. After scoring, feces were extensively homogenized using a Planetary Centrifugal "Thinky Mixer" ARM-310 (Thinky U.S.A., Inc., Laguna Hills, CA, USA), and portions were flash frozen in liquid nitrogen within 30 minutes of defecation for SCFA, IgA, ammonia, metabolomic, and microbiome analysis. Fecal homogenate that was not flash frozen was analyzed for moisture, ash, and minerals. Organic dry matter was calculated as 100%–(moisture % + ash %). Analysis of fecal moisture, ash, minerals, SCFA, metabolomics, and the composition of the fecal microbiome were carried out as previously described [28]. Fecal IgA was assessed using the feline IgA enzyme-linked immunosorbent assay quantitation set (Product #ab190547, AbCam; Cambridge, MA, USA) in which frozen fecal samples were lyophilized then mixed with extraction buffer prior to clarifying by centrifugation. Assay procedures were performed on collected supernatants according to the manufacturer’s recommendations. Fecal ammonia was assayed from frozen wet feces. Briefly, fecal material was extracted with 2 M KCl, centrifuged, and the supernatant was subjected to reaction with Berthelot’s reagent [33].

### Microbiome data processing

Microbiome raw sequence data were processed as previously described [28]. Briefly, FASTQ files were obtained from a MiSeq instrument (Illumina, San Diego, CA, USA) in pairs. The files were pre-processed into contigs, chimeras removed, and bacterial taxonomic classification was obtained using Mothur software [34] using a modified protocol of the MiSeq SOP [35] published on the Mothur website [36] according to Greengenes reference taxonomy [37]. Operational taxonomic units (OTUs) were identified based on taxonomic hierarchy. Sequences were deposited in the NCBI Sequence Read Archive under accession number PRJNA63518.

Alpha diversity indices were calculated on genus-level count data in the R programming environment [38] using the R vegan package [39]. Alpha diversity indices are presented as Hill numbers [40] of order “q”, corresponding to taxa richness (S; q = 0), exponential of the Shannon index (expH q = 1), and the inverse of the Simpson index (invSimp; q = 2). Respectively, these indices provide insight into microbiome community structure when taxa abundance is not considered (S), taxa contribute to the index according to their abundance (expH), and highly abundant taxa contribute to a greater degree to the diversity value (invSimp). Pielou’s Evenness (J) provides insight into the relative homogeneity of taxa abundance independent of the number of taxa detected [41]. In order to more fully assess the contribution of taxa abundance, Renyi alpha diversity series are reported, where increasing q denotes increasing influence of higher abundance taxa on diversity [42]. Beta diversity was calculated as 1-CqN (qβ) [43] and plotted on a continuum of increasing order q, in the same manner as for alpha diversity [44]. Renyi diversity series and qβ were generated with a custom R script [38] and are presented as curves for values of 0 < q < 10, calculated at intervals of q = 0.05. This script uses the R shiny package [45] and is available upon request from the corresponding author.

### Statistical analysis

For targeted endpoints, metabolomics and microbiome data the following statistical analyses were performed in JMP (versions 13.1–14.2. SAS Institute Inc., Cary, NC, 1989–2019): multivariate analysis of variance (MANOVA), independent t-test, and linear mixed modeling were employed to generate p values while partial least squares discriminant analysis (PLS-DA) was used to generate variable importance in projection (VIP) values using Nonlinear Iterative Partial Least Squares with 15 possible factors as the search space and K Fold cross validation (K = 7). Obtained metabolite and IgA values were log_2_-transformed and statistics were performed on transformed values. Categorical ordinal stool scores were approximated as a continuous variable. Genus-level OTU count data were centered natural log-ratio (CLR) transformed [46] to enable multivariate operations on compositional data [47] using the aldex.clr function in the R package ALDEx2 [48]. This operation consists of dividing the counts observed for an individual taxon by the geometric mean of counts for all observed taxa, followed by natural log transformation. Prior to CLR transformation, zero counts were inflated using Bayesian-multiplicative treatment [49] using the CZM attribute of the cmultRepl function in the R package zCompositions [50, 51]. For microbiome data, mixed modeling was carried out for weeks 3 and 6 separately with subject as random factor and using both week 3 and 6 timepoint data to test for an effect of RS across the entire study period with subject and collection number as random factors.

The sequential statistical process tested whether RS significantly impacted a given class of metabolites at either weeks 3 or 6, whether RS altered individual metabolites constituting as class at weeks 3 or 6, and finally whether there was an effect of RS on individual metabolites across the entire study. Determination of whether there was a difference by RS for a multivariate class of metabolites was performed separately at weeks 3 and 6 by MANOVA using the Identity function, which fits a model for each metabolite individually and then jointly tests the models together. Then, individual metabolites within a class were assessed at weeks 3 and 6 to determine which metabolites drove the significance observed for the class as a whole using t-tests when only one sample was analyzed per cat at each timepoint or linear mixed modeling when both collections at a timepoint were available. VIP scores were generated from PLS-DA and reported alongside p values at both weeks 3 and 6. To test for an effect of RS across the entire study period, linear mixed modeling was used with all data from both week 3 and 6 time points.

To account for the high dimensionality of the metabolite and genera-level microbiome data, false discovery-correcting q values were generated for all metabolites and genus level OTU in the R computing environment [38] using the “qvalue” function in the R package qvalue v2.14.1 [52] with separate vectors of p values from t-tests by timepoint and linear mixed modeling as input. Statistical significance was assigned to a metabolite or genera-level OTU where both of the following criteria were met: p ≤ 0.05 and false discovery rate q ≤ 0.1.

Global metabolome (log_2_-transformed) and microbiome (CLR-transformed) analyses were carried out on the Metaboanalyst platform v4.0 [53]. Orthogonal partial least squares discriminant analysis (OPLS-DA; which included all 736 observed metabolites or 252 identified genus-level OTUs) with 2000 permutations as well as sparse partial least squares (SPLS; which included 20 metabolites or OTUs after dimensionality reduction), two components and 5-fold cross validation were carried out to detect metabolome- and microbiome-wide differences between HRS versus LRS-fed cats separately at weeks 3 and 6. Random forest was performed separately at each timepoint and in order to detect metabolite and genus-level OTU predictors of group identity (random forest number of trees = 2000, number of predictors = 10, randomness = on).

## Results

### Food production and analysis

Identically formulated foods (Table 1) were produced with either high or low shear extrusion force (S1 Fig). Percent cook analysis indicated that greater concentrations of RS were present in the low shear food (percent cook: high shear = 98.4%; low shear = 72.2%). Analysis of viscosity indicated a peak viscosity (indicative of cooked starch) of 1389 cP for high shear and 1009 cP for low shear, while final viscosity (which increases with higher molecular weight starch) was 1241 cP for high shear and 1780 cP for low shear. Together, these results indicate that the low shear extrusion conditions led to a food that contained less cooked starch with likely more RS and that its starch content was of a higher molecular weight. Hereafter, the foods produced by high and low shear are designated as low and high RS (LRS and HRS), respectively.

There were no nutritionally meaningful differences between the LRS and HRS foods in moisture, fat, protein, crude fiber, ash, or gross energy; neutral detergent fiber was marginally decreased in the HRS food (Table 1).

### Study animals, food intake, and weight

Forty domestic short-haired breed cats were included in the study and split evenly between the LRS and HRS food groups, with a mean (SE) age of 8.9 (0.5) years (S1 Table). No stools were successfully collected from four cats during the monitoring periods at either weeks 3 or 6, and these cats were necessarily excluded from analysis.

### Food digestibility and intake

There was no difference between the LRS and HRS foods for intake of calories (*P* = 0.191) or grams of food (*P* = 0.347) (S2 Table). There was also no difference between groups for intake of total starch, protein, or fat, nor was body weight changed from baseline for either group.

Results from pre-trial AAFCO quantitative collection protocol digestibility studies for each food showed no significant differences in digestibility by extrusion type, though the mean of each digestibility parameter was numerically lower for the HRS food (Table 1). Stool scores are measured as part of the digest study on a 5-point scale; moisture is also measured. In these preliminary digest studies, stool scores were not different when feeding HRS and LRS foods (stool score mean ± SD: LRS, 4.25 ± 0.53; HRS, 4.57 ± 0.81; *P* = 0.437 by independent t-test). As well, stool moisture levels were not different between HRS- and LRS-fed cats in the preliminary digest studies (moisture mean ± SD: LRS, 60.9 ± 5.0; HRS, 64.8 ± 5.8; *P* = 0.244 by independent t-test). Thus, both subjective, blinded stool scoring and objective fecal moisture data from the preliminary digest trials indicated that RS feeding at the levels employed herein did not meaningfully affect stool firmness.

### Stool scores and proximate analyses

Similar to the results of the preliminary digest studies, in the main feeding trial there was no difference in stool scores between the cats consuming LRS versus HRS foods at either week 3 (mean [SE], 4.81 [0.09] versus 4.69 [0.11], respectively; *P* = 0.53) or week 6 (4.94 [0.04] versus 4.84 [0.08], respectively; *P* = 0.89). There were, however, small but statistically significant increases in moisture (3.1−4.5% increased in HRS-fed cat feces, *P* = 0.007) offset by decreased organic dry matter in the feces of cats fed the HRS food (2.3−3.0% decreased, *P* = 0.005) (S3 Table). Taken together, stool scores and fecal moisture analyses showed little change with increased RS feeding in HRS-fed cats. The only fecal mineral consistently altered at both timepoints was potassium, which was increased in feces from HRS-fed cats, perhaps indicating a compensatory colonic osmoregulatory response to increased RS.

### Fecal global metabolomics

Metabolomic analysis performed on fecal samples taken at weeks 3 and 6 identified 736 metabolites. Of these, 47% were significantly different (*P* ≤ 0.05, q ≤ 0.1) between the low and HRS foods by mixed modeling across both timepoints (36% increased and 11% decreased in the HRS food) (S3 Table). At week 3, 43% of metabolites had a PLS-DA VIP score > 1.0 (minimum root mean predictive residual sum of squares = 0.9, minimizing number of factors = 11, cumulative Q2 = 0.94, cumulative R^2^Y = 0.99) while at week 6, 41% of metabolites had a PLS-DA VIP score > 1.0 (minimum root mean predictive residual sum of squares = 0.41, minimizing number of factors = 3, cumulative Q2 = 0.98, cumulative R^2^Y = 0.97). The significant metabolite differences between the LRS and HRS-fed groups at week 3 were highly persistent to week 6 of feeding (week 3 log_2_FC versus week 6 log_2_FC Pearson correlation; adjusted R^2^ = 0.91, *P* < 0.001, slope = 0.99).

**Fig 1:**
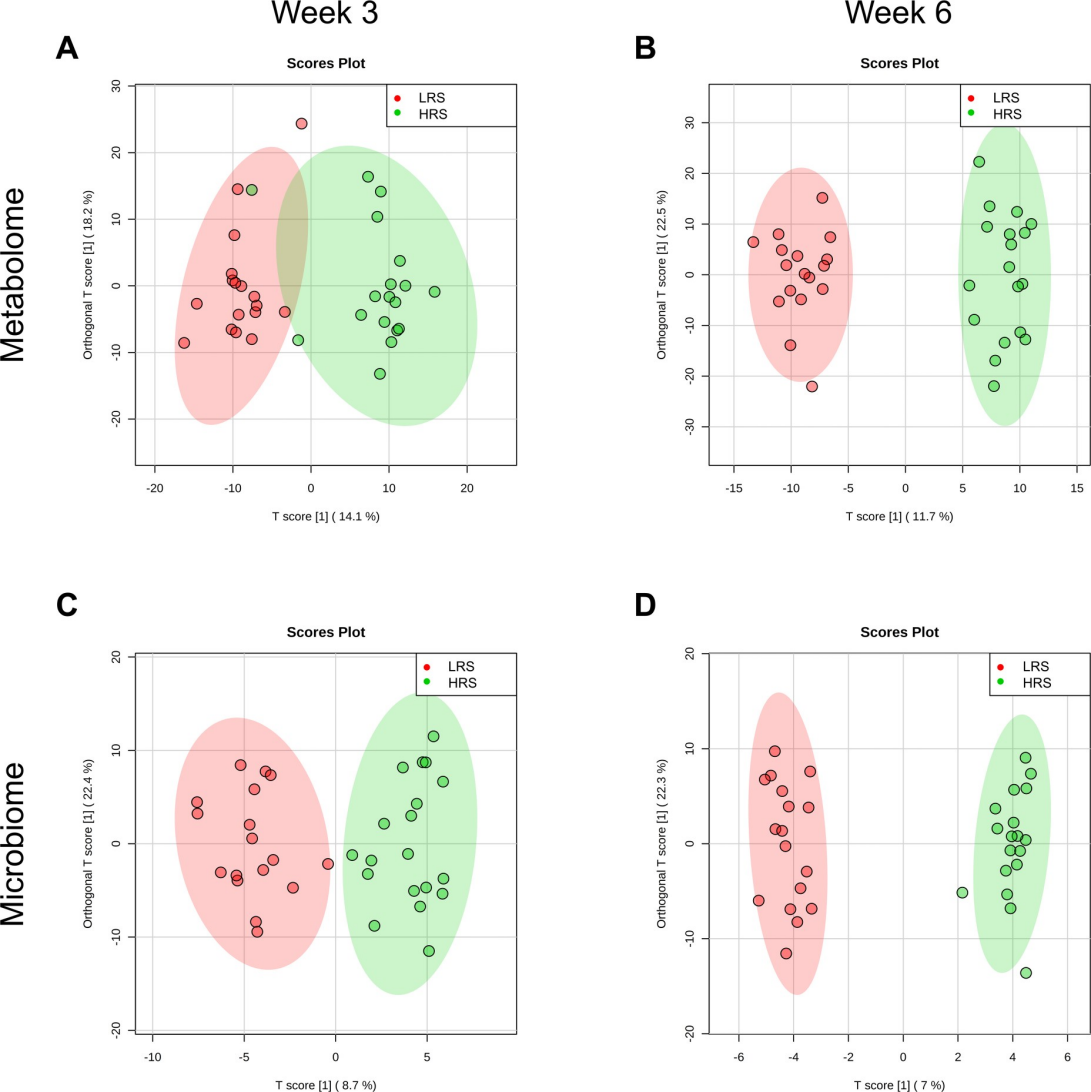
Orthogonal partial least squares analysis of whole fecal differences at weeks 3 and 6 between LRS and HRS food-fed cats for the metabolome (A,B) and microbiome (C,D). Shading indicates 95% confidence regions. Metabolome permutation statistics at week 3 were: Q2 = 0.38, *P* < 0.001; R^2^Y = 0.80, *P* = 0.014 and at week 6 were: Q2 = 0.86, *P* < 0.001; R^2^Y = 0.96, *P* < 0.001. Microbiome permutation statistics at week 3 were: Q2 *P* < 0.001; R2Y *P* = 0.059 and at week 6 were: Q2 *P* < 0.001; R2Y *P* = 0.056. HRS, high resistant starch; LRS, low resistant starch.

OPLS analysis indicated greater metabolome-wide differences between cats fed LRS and HRS foods at week 6 than week 3; T-Scores and orthogonal T-Scores explained approximately 33% of variation at both weeks (Fig 1A and 1B). Dimensionality reduction of the number of discriminatory analytes from 736 to 20 using SPLS analysis on two components provided complete separation (S2 Fig; SPLS loadings are in S4 Table). Random forest analysis provided discrimination between the LRS and HRS food-fed cats at weeks 6 and 3 (overall out of bounds class error 5.7% and 17.1%, respectively; S3 Fig).

Based on the known actions of gut microbiota on undigested macronutrients, as well as prominence in the global metabolomics analysis, metabolites of carbohydrates, proteins, and lipids were further analyzed. Results in total show that most metabolite features of these nutrient classes were significantly different by dietary RS concentration (Table 2).

**Table 2:** Summary of metabolite class changes.

Metabolism Type	Class	HRS/LRS
**Saccharolysis**	RS sugars	****↑****
	other sugars	****↑****
Fermentation	SCFA	****↑****
	lactate	****↑****
**Proteolysis**	dipeptides	****↑****
	amino acids	****↑****
Putrefaction	ammonia	**↓**
	beneficial indoles	****↑****
	detrimental indoles	**no change**
	biogenic tryptophan amine	**↓**
	kynurenine	**no change**
	serotonin	**↓**
	polyamines	****↑****
**Lipolysis**	1-acylglycerols	**↓**
	lysophospholipids	****↑****
	fatty acids	****↑** (saturated) ↓ (unsaturated)**
	ketone body metabolism	****↑****
Bioactive lipids	N-acylethanolamines	**↓**
	2-acylglycerols	**↓**
	NAD(H) precursors	****↑****

HRS, high resistant starch; LRS, low resistant starch; NAD(H), nicotinamide adenine dinucleotide, oxidized or reduced form; SCFA, short-chain fatty acid.

### Saccharolysis and fermentation

#### RS- and epithelial-derived saccharides

Maltotriose, maltose, and glucose, known to be derived from microbial saccharolysis of RS, were increased in HRS-fed cats, but glucose was not increased at week 6 (Table 3). No RS-derived sugars were ever higher in the LRS group. Other, non-RS derived sugars increased in the HRS group (Table 3) including sugars typically associated with epithelial surfaces (mannose, N-acetylglucosaminylasparagine, glucuronate). No oligo- or monosaccharides were observed to be higher in the LRS group at either timepoint.

**Table 3:** Sugars derived from resistant starch and other sugars detected via metabolomic screening in fecal samples taken at weeks 3 and 6 from cats fed LRS or HRS food.

Type	Metabolite	Week 3	Week 6
		Log2(FC)	Log2(FC)
**RS sugar**	glucose		
	maltose		
	maltotriose		
	maltotetraose		
**Cell surface and plant sugar**	mannose		
	N-acetylglucosaminylasparagine		
	glucuronate		
	diacetylchitobiose		
	fucose		
	arabinose		
	erythrose		
	fructose		
	ribose		
	ribulose/xylulose		
	sedoheptulose		
	xylose		
	erythronate		
	threonate		

FC, fold-change of HRS/LRS; HRS, high resistant starch; LRS, low resistant starch.

Concentrations of a given metabolite in the HRS group versus the LRS group by independent t-test (week 3 or week 6) or mixed model (weeks 3 & 6) at *P* < 0.05 and q < 0.1 are shown as significantly higher (red), significantly lower (green), or unchanged (white). Full numerical data are available in S3 Table.

#### SCFA production

At both weeks, the concentrations of the straight-chain SCFA butyrate and the ratio of total SCFA to total branched-chain SCFA were significantly higher in fecal samples from cats that consumed the HRS food (Table 4).

**Table 4:** Short-chain fatty acids in fecal samples taken at weeks 3 and 6 from cats fed LRS or HRS food.

	Week 3				Week 6			
	LRS	HRS	HRS-LRS	Independent t-test	LRS	HRS	HRS-LRS	Independent t-test
	Mean (SE)	Mean (SE)	Delta	p	Mean (SE)	Mean (SE)	Delta	p
Fatty acid								
Acetate	2781.2 (266.7)	3456.8 (269.6)	675.6	0.087	2378.8 (240.1)	2681.9 (193.1)	303.1	0.338
Propionate	1289.8 (107.8)	1460.8 (114.8)	171.0	0.288	1033.1 (112.6)	1165.1 (92.4)	132.0	0.377
Butyrate*	1369.0 (120.9)	2397.8 (183.7)	1028.7	< 0.001	1303.8 (93.2)	1842.8 (171.6)	539.0	0.013
Isobutyrate	282.0 (25.6)	268.6 (20.2)	-13.4	0.685	281.6 (20.1)	250.9 (17.7)	-30.7	0.265
2-methylbutyrate	176.8 (17.4)	169.4 (12.0)	-7.4	0.730	185.8 (17.5)	165.8 (13.6)	-19.9	0.380
Isovalerate	342.7 (29.3)	339.1 (22.8)	-3.6	0.923	350.4 (24.2)	310.0 (20.2)	-40.4	0.216
Total SCFA	5440.0 (444.4)	7315.4 (422.3)	1875.4	0.005	4715.7 (396.8)	5689.7 (359.2)	974.1	0.084
Total BCFA	801.4 (71.9)	777.0 (54.2)	-24.4	0.789	817.7 (61.4)	726.8 (50.9)	-91.0	0.268
SCFA/BCFA	7.0 (0.5)	9.9 (0.8)	2.9	0.004	5.9 (0.5)	8.2 (0.7)	2.3	0.014

*Two subjects were removed from analysis as statistical outliers: values of 4229.4 (Subject 14) and 3107.2 ppm (Subject 35).

BCFA, branched -chain fatty acid; FC, fold change; HRS, high resistant starch; LRS, low resistant starch; SCFA, short-chain fatty acid; SE, standard error.

Assays were performed as previously described [28].

Five of the six straight- and branched-chain SCFAs quantified by targeted analysis were also observed as glycine conjugates in the metabolomics dataset (S3 Table); unconjugated SCFAs are typically not directly observed in metabolomic screens due to their volatility. Glycine conjugates of SCFA all increased while the BCFA conjugates isobutyrylglycine and isovalerylglycine decreased or were unchanged, respectively.

### Proteolysis and putrefaction

#### Dipeptides and amino acids

Fecal concentrations of dipeptides, incomplete products of proteolysis, were significantly different as a class according to dietary RS concentration. There were 9/24 observed dipeptides significantly increased in the feces of HRS-fed cats across the entire study (Table 5). Amino acids were higher in the HRS group, an effect that increased from week 3 to 6 (Table 5). No dipeptides or amino acids were higher in the LRS group relative to the HRS group at either timepoint.

**Table 5:** Dipeptides and amino acids detected via metabolomic screening in fecal samples taken at weeks 3 and 6 from cats fed LRS or HRS food.

Type	Metabolite	Week 3	Week 6
		Log2(FC)	Log2(FC)
**Dipeptide**	alanylleucine		
	cysteinylglycine		
	glutaminylleucine		
	glycylisoleucine		
	glycylleucine		
	glycylvaline		
	histidylalanine		
	isoleucylglycine		
	leucylalanine		
	leucylglutamine		
	leucylglycine		
	lysylleucine		
	phenylalanylalanine		
	phenylalanylglycine		
	prolylglycine		
	prolylhydroxyproline		
	threonylphenylalanine		
	tryptophylglycine		
	tyrosylglycine		
	valerylphenylalanine		
	valeryltryptophan		
	valylglutamine		
	valylglycine		
	valylleucine		
**Amino acid**	alanine		
	arginine		
	asparagine		
	aspartate		
	cysteine		
	glutamate		
	glutamine		
	glycine		
	histidine		
	isoleucine		
	leucine		
	lysine		
	methionine		
	phenylalanine		
	proline		
	serine		
	taurine		
	threonine		
	tryptophan		
	tyrosine		
	valine		

FC, fold change; HRS, high resistant starch; LRS, low resistant starch.

Concentrations of a given metabolite in the HRS group versus the LRS group by independent t-test (week 3 or week 6) or mixed model (weeks 3 & 6) at *P* < 0.05 and q < 0.1 are shown as significantly higher (red), significantly lower (green), or unchanged (white). Full numerical data are available in S3 Table.

#### Ammonia

Fecal ammonia was significantly decreased at week 6 in the HRS group (log_2_ FC = -0.30, *P* = 0.01) but not at week 3 (S3 Table). Leucine metabolites isocaproate and isovalerate did not differ across groups, indicating that ammonia-generating Stickland fermentation was minimally affected by dietary RS concentration [54] (S3 Table). Similarly, increases in glycine betaine and sarcosine (S3 Table), both electron acceptors in Stickland reactions, were in opposition of those predicted if ammonia accumulation was substantial through this route [55].

#### Indoles

Indoles, putrefactive metabolites of tryptophan, were significantly different by RS at both weeks 3 and 6 (Table 6 and S3 Table). Notable were increases in indolelactate and indolepropionate. In contrast, indolin-2-one was decreased, and indoles hydroxylated on the aromatic ring as well as their sulfate conjugates were unchanged. Additional routes of metabolic disposal of tryptophan by gut microbes include the biogenic amine tryptamine, the kynurenine pathway, and serotonin production. Tryptamine and serotonin were both decreased in the feces from the HRS group, while kynurenines did not differ between groups (S3 Table).

**Table 6:** Indoles and polyamines detected via metabolomic screening in fecal samples taken at weeks 3 and 6 from cats fed LRS or HRS food.

Type	Metabolite	Week 3	Week 6
		Log2(FC)	Log2(FC)
**Indole**	2-oxindole-3-acetate		
	3-hydroxyindolin-2-one		
	3-hydroxyindolin-2-one sulfate		
	3-indoxyl sulfate		
	5-hydroxyindoleacetate		
	indole		
	indoleacetate		
	indoleacetylglycine		
	indolelactate		
	indolepropionate		
	indolin-2-one		
	methyl indole-3-acetate		
**Polyamine**	cadaverine		
	N-acetyl-cadaverine		
	putrescine		
	N-acetylputrescine		
	N-acetyl-isoputreanine		
	spermidine		
	diacetylspermidine		
	(N(1) + N(8))-acetylspermidine		
	N(1)-acetylspermine		
	N1,N12-diacetylspermine		
	carboxyethyl-GABA		

FC, fold change; GABA, gamma aminobutyric acid; HRS, high resistant starch; LRS, low resistant starch.

Concentrations of a given metabolite in the HRS group versus the LRS group by independent t-test (week 3 or week 6) or mixed model (weeks 3 & 6) at *P* < 0.05 and q < 0.1 are shown as significantly higher (red), significantly lower (green), or unchanged (white). Full numerical data are available in S3 Table.

#### Polyamines

Polyamines are products of microbial putrefaction of amino acids lysine, arginine, agmatine, and ornithine, with methylthioadenosine produced as a byproduct. Polyamines and their catabolic intermediates were increased in the feces from HRS-fed cats at week 3 but not at week 6 (Table 6). The primary polyamines cadaverine (lysine-derived) and putrescine (arginine-, ornithine-, or agmatine-derived) were significantly increased in the HRS group but spermidine was unchanged; spermine was not observed in the dataset. Although there were extensive increases in free amino acids in the HRS group (*vide supra*), neither arginine nor lysine precursors to polyamines were increased. Agmatine was not different by RS, but ornithine was increased in the HRS group (S3 Table). Methylthioadenosine, a byproduct of putrescine chain elongation, was increased in the HRS group (S3 Table).

### Lipolysis and production of bioactive lipids

#### Lipolysis

Incomplete products of lipolysis were found to vary by RS at both timepoints; 1-monoacylglycerols increased while lysophospholipids decreased. Lysophospholipids derived from all three phospholipids (phosphatidylethanolamine, phosphatidylcholine, and phosphatidylserine) and containing both saturated (palmitoyl, stearoyl) as well as unsaturated (oleoyl, linoleoyl) acyl chains were increased in the feces from HRS-fed cats (S3 Table). Complete products of lipolysis, non-esterified fatty acids, varied by RS concentration depending on their chain length (S3 Table); the specific effects of RS feeding on long-chain polyunsaturated fatty acids were quite prominent. Of the six omega-3 (n3) NEFA observed in the dataset, four were persistently decreased in the HRS group at both weeks. Conversely, medium-chain saturated fats valerate, caproate, and heptanoate were increased by HRS feeding; since these are not present at significant amounts in the diet, they are likely derived from β-oxidation of longer chain dietary fatty acids.

#### Ketogenesis

At both weeks 3 and 6, β-hydroxybutyrate (BHB) was increased in feces from HRS-fed cats (Table 7). Caproate (hexanoate), converted to BHB via the intermediacy of butyrate through β-oxidation by gut microbiota, was increased in feces from HRS-fed cats at both timepoints and the product of its β-oxidation, 3-hydroxyhexanoate, was also increased by mixed modeling across the study. As indicated above, the SCFA butyrate can undergo β-oxidation to form BHB, and this SCFA was increased in the HRS group. Together, these data indicate that a ketogenic state is potentially induced in the colonic lumen with RS feeding in cats.

**Table 7:** Ketogenic metabolites, ethanolamides, and bile acids detected via metabolomic screening in fecal samples taken at weeks 3 and 6 from cats fed LRS or HRS food.

Type	Metabolite	Week 3	Week 6
		Log2(FC)	Log2(FC)
**Ketogenesis**	3-hydroxybutyrate (BHB)		
	3-hydroxy-3-methylglutarate (HMG)		
	caproate (6:0)		
	3-hydroxyhexanoate		
**Cannabinoid ethanolamide**	palmitoyl ethanolamide		
	palmitoleoyl ethanolamide		
	margaroyl ethanolamide		
	stearoyl ethanolamide		
	oleoyl ethanolamide		
	linoleoyl ethanolamide		
	dihomo-linolenoyl ethanolamide		
	arachidoyl ethanolamide (20:0)		
	arachidonoyl ethanolamide		
	behenoyl ethanolamide (22:0)		
	erucoyl ethanolamide (22:1)		
	lignoceroyl ethanolamide (24:0)		
	nervonoyl ethanolamide (24:1)		
**Bile acid**	chenodeoxycholate		
	cholate		
	cholate sulfate		
	glycochenodeoxycholate		
	hyocholate		
	taurochenodeoxycholate		
	taurocholate		
	taurocholenate sulfate		
	ursocholate		
	12-dehydrocholate		
	3-dehydrocholate		
	6-oxolithocholate		
	7-ketodeoxycholate		
	7-ketolithocholate		
	dehydrolithocholate		
	deoxycholate		
	glycodeoxycholate		
	glycolithocholate		
	isohyodeoxycholate		
	isoursodeoxycholate		
	lithocholate		
	taurodeoxycholate		
	taurolithocholate 3-sulfate		

FC, fold change; HRS, high resistant starch; LRS, low resistant starch.

Concentrations of a given metabolite in the HRS group versus the LRS group by independent t-test (week 3 or week 6) or mixed model (weeks 3 & 6) at *P* < 0.05 and q < 0.1 are shown as significantly higher (red), significantly lower (green), or unchanged (white). Full numerical data are available in S3 Table.

#### Endocannabinoids

N-acylethanolamine endocannabinoids are products of mammalian and perhaps microbial metabolism. These cannabinoids were significantly decreased by RS feeding (Table 7), with an effect greater for the very long chain species. While 2-monoacylglycerols were decreased by RS feeding at 3 weeks, these transient effects contrasted with the persistent effect on very long-chain N-acylethanolamines.

#### Bile acids

As a multivariate class, bile acids significantly differed by RS at weeks 3 and 6 (S3 Table). Of the 23 primary and secondary bile acids detected in the fecal metabolomics data, 12 were increased in the feces from HRS-fed cats and only three decreased relative to the LRS group by mixed modeling across timepoints (Table 7). The effect for individual bile acids was similar across weeks 3 and 6; however, there was a trend toward decreased impact of RS on bile acids at the week 6 timepoint. The largest magnitudes of changes were increases in the primary bile acids cholate and taurocholenate sulfate and the secondary bile acids 12-dehydrocholate, 3-dehydrocholate and a decrease in the secondary bile acid 6-oxolithocholate. The secondary bile acid deoxycholate was also significantly decreased across the study duration.

### NADH-coupled hydroxyl:Oxo redox ratios

The ratio of NADH:NAD^+^-coupled hydroxyl:oxo redox pairs derived from gut microbial metabolism of both dietary carbohydrate and protein catabolism were increased in the HRS group, with a larger effect at week 6 than 3 (Table 8). The increased hydroxyl:oxo ratio stemmed from elevated concentrations of the reduced (hydroxyl) member of the redox couple; the oxidized (oxo) member of the redox couple did not differ across groups. This held true for redox couples derived from the microbiome-mediated metabolism of sugars and the amino acids isoleucine, leucine, valine, phenylalanine, as well as for hydroxyl metabolites of tryptophan metabolism indolelactate, imidazole lactate, and 3-(4-hydroxyphenyl)lactate (S3 Table). Since the relative concentrations of the hydroxyl:oxo forms of these redox ratio pairs are in equilibrium with the NADH:NAD^+^ redox couple, we examined the concentrations of all observed fecal metabolites containing a nicotinic moiety (S3 Table). Changes of the class related to NADH metabolism [56] were among the largest seen in this study and were also highly persistent across timepoints.

**Table 8:** Redox-coupled congeners detected via metabolomic screening in fecal samples taken at weeks 3 and 6 from cats fed LRS or HRS food.

Source	Redox Ratios and Congeners	Week 3	Week 6
		Log2(FC)	Log2(FC)
Sugar	lactate/pyruvate*		
	lactate		
	pyruvate		
Isoleucine	2-hydroxy-3-methylvalerate/3-methyl-2-oxovalerate*		
	2-hydroxy-3-methylvalerate		
	3-methyl-2-oxovalerate		
Leucine	alpha-hydroxyisocaproate/4-methyl-2-oxopentanoate*		
	alpha-hydroxyisocaproate		
	4-methyl-2-oxopentanoate		
Valine	alpha-hydroxyisovalerate/3-methyl-2-oxobutyrate*		
	alpha-hydroxyisovalerate		
	3-methyl-2-oxobutyrate		
Phenylalanine	phenyllactate/phenylpyruvate*		
	phenyllactate (PLA)		
	phenylpyruvate		

*Derived ratios were not included in q value estimation.

FC, fold change; HRS, high resistant starch; LRS, low resistant starch.

Concentrations of a given metabolite in the HRS versus the LRS group by independent t-test (week 3 or week 6) or mixed model (weeks 3 & 6) at *P* < 0.05 and q < 0.1 are shown as significantly higher (red), significantly lower (green), or unchanged (white). Full numerical data are available in S3 Table.

### Fecal microbiome

A total of 406 OTUs were classified at the genus level in the fecal samples, including many with unknown genus identification. Of these, 252 OTUs were identified at the genus level and subjected to univariate statistical analysis by OTU; 30% were different for at least one time point and 21% were different across the study (S3 Table). Fold changes (FC) for LRS vs HRS present at week 3 were highly persistent to week 6 (week 3 ln[FC] versus week 6 ln[FC] Pearson correlation; adjusted R^2^ = 0.92, *P* < 0.001, slope = 1.11). Mixed modeling assessment using data from both timepoints showed that changes in fecal OTUs that were significant at only one of the timepoints were supported by trends at the other timepoint.

Subsequent to Aitchison transformation with the CLR function [47], a global microbiome assessment was carried out. OPLS assessment of the CLR-transformed 252 genus-level classified OTUs showed complete separation of the 95% confidence regions of the HRS versus LRS groups at both weeks 3 and 6. The T-Score and orthogonal T-Score explained approximately 30% of variation at both timepoints (Fig 1C and 1D). Dimensionality reduction of the number of analytes using SPLS analysis provided separation of the 95% confidence regions at both weeks 3 and 6 and accounted for approximately 24% and 15% of variation, respectively (S2 Fig). There were nine OTUs in common in component 1 of both timepoints, indicating that these genera were persistently discriminatory for RS consumption in cats. Among these nine OTUs were saccharolytic (*Lactobacillus*) and proteolytic (*Peptococcus*, *Peptostreptococcus*) bacteria as well as methanogenic Archaea (*Methanosarcina*) (S4 Table). Random forest analysis provided a marginally better classification of the groups at week 6 than week 3 (overall out of bounds class error, 14.3% and 19.4%, respectively; S4 Fig). Members of phylum Firmicutes were prominent predictors of dietary RS concentration; at both weeks 3 and 6, eight of the top 20 ranked OTU were genera in this phylum, while members of phylum Bacteroidetes only accounted for 3/20 top ranked features. There were 11 genera in common and six of these were from phylum Firmicutes while only two were from phylum Bacteroidetes in the union of the 3 and 6 week random forest classifier data. The genera from phylum Firmicutes had both saccharolytic (*Lactobacillus*, *Blautia*, *Megasphaera*) and proteolytic (*Peptococcus*, *Peptostreptococcus*) proclivities. When assessed at the phylum level, the Bacteroidetes:Firmicutes ratio was significantly lower in the microbiomes of the HRS group across the study and at each timepoint (S3 Table). Analysis at the phylum level also showed that the most strongly increased phylum in the HRS group was Acidobacteria, which appears to be consistent with the observation of increased fecal lactic acid (lactate) in this group.

**Fig 2:**
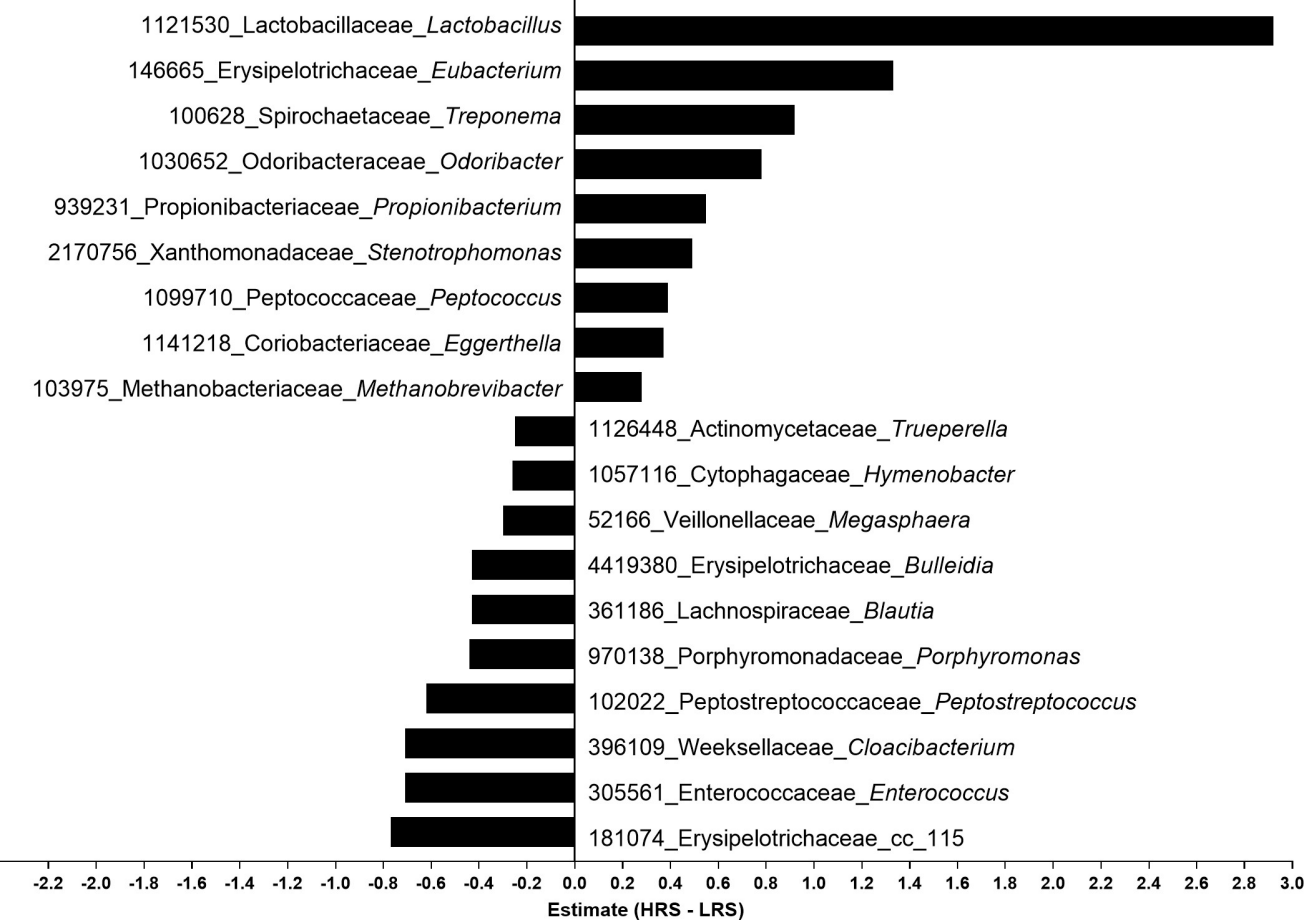
Significant genus-level taxa differences in the fecal microbiome from consumption of HRS or LRS food using a mixed model of weeks 3 and 6. Subject and collection number were random factors. Operational taxonomic unit number, family, and genus are shown for those with the greatest differences; full data are available in S3 Table. HRS, high resistant starch; LRS, low resistant starch.

The most prominent differences in individual taxa by mixed modeling were decreases in the genera *Enterococcus*, *Peptostreptococcus*, and *Megasphaera*, while *Porphyromonus*, *Acidaminococcus*, *Blautia*, *Ruminococcus*, and *Peptoniphilus* were decreased less consistently across the study. Prominently increased genera were *Lactobacillus*, *Eubacterium*, *Odoribacter*, *Treponema*, *Stenotrophomonas*, *and Peptococcus* (Fig 2; S3 Table). Thus, abundances of genera associated with both saccharolytic and proteolytic process were markedly altered by consumption of RS in the HRS food, consistent with the observed changes to both saccharolytic/fermentative and proteolytic/putrefactive metabolites.

Community structure was assessed, using all 406 OTUs classified at the genera level, by way of diversity indices based on the total number of observed taxa as well as relative abundances of those taxa. Analysis of alpha diversity showed that there was persistently higher diversity in the fecal microbiome of cats consuming the HRS versus LRS foods in a manner that persisted across the timepoints (Table 9). Taxa richness (S) was significantly higher in the HRS group at both weeks 3 and 6. The exponent of Shannon index (expH) and the inverse of Simpson index (invSimp) were also increased at weeks 3 and 6. Pielou’s Evenness (J) was not different at either timepoint, although there was a trend towards increased evenness when assessed across both timepoints (*P* = 0.073). The percent mean increase in diversity of the HRS group from the LRS group for both the Shannon and Simpson diversity indices was approximately the same (~24% at 3 weeks, ~20% at 6 weeks for both expH and invSimp). To better gain insight into the degree to which taxa abundance impacted observed differences in alpha diversity, a sweep of diversity values from 0 < q < 10 at intervals of q = 0.05 was carried out using data from the week 3 and 6 timepoints. Mixed modeling indicated that the HRS group manifested higher alpha diversity until q = 5, beyond which there were not significant differences by shear type (S5 Table). Beta diversity (1-CqN; qβ) was assessed along the same continuum of order q (0 < q < 10), and the qβ curve of HRS-fed cats was markedly higher than that of the LRS group in the range of 2.5 < q < 8 (S5 Table).

**Table 9:** Alpha diversity metrics from fecal samples from cats fed LRS or HRS food.

Alpha Diversity Index	Week 3				Week 6			
	LRS	HRS	HRS-LRS	Mixed Model*	LRS	HRS	HRS-LRS	Mixed Model*
	Mean (SE)	Mean (SE)	Delta	p value	Mean (SE)	Mean (SE)	Delta	p value
S	104.63 (2.44)	109.42 (1.90)	4.78	0.004	102.85 (3.21)	110.66 (2.76)	7.81	0.004
expH	16.92 (0.79)	21.06 (0.84)	4.15	0.027	16.98 (0.67)	20.50 (0.66)	3.52	0.034
invSimp	10.04 (0.51)	12.34 (0.68)	2.30	0.003	10.34 (0.50)	12.21 (0.53)	1.87	0.013
J	0.60 (0.01)	0.64 (0.01)	0.04	0.221	0.61 (0.01)	0.64 (0.01)	0.03	0.099

*Subject as a random factor. ^**†**^ Subject and collection number as random factors.

expH, exponential of the Shannon index; HRS, high resistant starch; invSimp, inverse of the Simpson index; J, Pielou's evenness; LRS, low resistant starch; S, taxa richness; SE, standard error.

Data are presented as mean (SE).

### Fecal secretory IgA

In order to assess the degree to which RS feeding impacted the host-microbiome mutualistic relationship, IgA was assessed in feces at week 6. Fecal IgA concentrations from cats fed the HRS food were significantly higher at week 6 compared with those fed the LRS food (S3 Table).

## Discussion

This study examined the effects of feline consumption of RS produced through different extrusion conditions on gut microbiome metabolism of dietary carbohydrate, protein, and fat. The starch in the HRS food was 27% less cooked as assessed by independent methods (percent cook and viscosity analysis) and its molecular weight was 40% higher, the result of a 60% reduction of extrusion shear force. The effect of RS consumption on feline gut microbiome metabolism was pervasive and persistent; nearly 50% of the fecal metabolome was altered and these differences were highly correlated across time points. There was no overt effect on stool firmness as assessed by stool quality score and fecal moisture, and stool macro and mineral analyses were minimally impacted, indicating the feasibility of intervening with dietary RS in domestic cats to produce sizeable shifts in microbiome function without producing wide deviations in stool firmness or composition.

In the current study, RS consumption provided carbohydrates for gut microbial saccharolysis and fermentation. RS-derived substrates for saccharolysis were increased in feces alongside the saccharolytic products of epithelial cell polysaccharides [57, 58]. There was a greater effect as time progressed perhaps indicating adaptation to RS consumption. On a molar basis, RS is a particularly efficient precursor of butyrate production [59] and has been shown to ameliorate proteinuria in a mouse model of CKD [60], aspects that may be beneficial to diabetic cats, which have a paucity of butyrate-producing bacteria in their microbiome [61]. Increased fecal butyrate in the HRS diet group indicates the potential for dietary RS to exert a benefit in cats. Dietary RS is also a known source of lactate production by lactic acid bacteria, with the lactate used for subsequent production of butyrate [62, 63]. Increased lactate in the current study in HRS-fed cats may have contributed to the increased concentration of butyrate that was observed. Bacteria complexed with IgA, which is secreted into the luminal space of the colon and binds to gut commensals, promote gut homeostasis and induce a more robust and pervasive effect on host physiology than uncomplexed bacteria [64, 65]. Dietary RS consumption increases IgA in rats [66, 67], mice [68], and pigs [69], an effect that may be driven by SCFA production [70].

Proteolytic and putrefactive metabolites of protein also appeared in feces. Undigested protein delivered to the colon is subject to microbial proteolysis, and postbiotics derived from putrefaction of amino acids are reabsorbed into systemic circulation [71]. Dietary RS increased concentrations of dipeptide and amino acid products of proteolysis. However, ammonia, which is a marker of microbial amino acid putrefaction [72], decreased in the HRS-fed cats. Products of ammonia-generating Stickland fermentation were not different by RS concentration, while concentrations of electron acceptors for these reactions indicated reduced activity through this pathway. This may indicate that the increased carbohydrate availability and fermentation with RS feeding lessened microbial putrefaction, as has been previously observed [73], and that putrefaction to generate ammonia [21] was decreased by RS feeding.

Products of amino acid putrefaction are associated with detrimental health consequences, particularly for renal-impaired individuals [74, 75], also demonstrated in cats [76]. However, protein escaping digestion does not necessarily have negative consequences to health [77] and can provide benefits [78–80]; undigested protein has previously been shown to increase gut microbial butyrate and IgA [81, 82], as well as heighten the effect of dietary RS to increase microbial production of SCFA [83]. Gut microbial metabolites of tryptophan [23, 84, 85] have beneficial health effects on gut and systemic physiology [77]. In the current study, higher RS increased fecal indole-3-propionate, a gut microbe metabolite of tryptophan that improves gut barrier integrity [86] and interferes with metabolism by pathogens [87]. Indole-3-lactate, produced by *Bifidobacterium* species [88], has anti-inflammatory activity [89], and is converted to indole-3-propionate, was also increased in the HRS-fed cats. Previous reports show RS feeding increased indole-3-lactate and indole-3-acetate in serum of CKD rats, although these were decreased or unchanged in cecal contents [90]. Tryptophan-derived biogenic amine tryptamine and serotonin were decreased by RS feeding while kynurenines were unchanged. Serotonin can influence gut motility and thus transit time of food and stool firmness. Commensal microbiota partially determine concentrations of colonic serotonin in an SCFA-dependent manner by direct synthesis [91], stimulation of release from enterochromaffin cells and by modulating reuptake by serotonin transporters [92]. Though the cats enrolled in this study were healthy, and stool firmness was not impacted by RS feeding, the finding that RS feeding reduces fecal serotonin concentrations in cats may hold promise for recommendation of diets for management of feline subjects with chronic watery stools.

Gut microbiota synthesize polyamines from arginine and its derivatives as well as lysine, cadaverine, putrescine, and spermidine [1, 93]. Microbial production of polyamines abrogates the detrimental impact of aging on intestinal epithelia [94, 95] and may serve protective roles against endothelial dysfunction [96] and neoplastic processes [97]. Cadaverine, putrescine, and acetylated forms of spermidine were increased by HRS food although whether cats may benefit from RS through increased polyamine production remains to be tested.

Although fat is typically highly digestible and absorbed in healthy subjects, products of partial and complete lipolysis were observed in feces alongside bioactive lipids. The ratio of dietary fat to starch has been shown to influence the microbiome composition and function in dogs [98]. Dietary fat that escapes digestion is available in the colon as a nutrient source for resident bacteria [99], and gut commensals harbor capacity for degradation of fatty acids [100] via β-oxidation [101]. Gut microbial β-oxidation converts longer chain fatty acids to SCFA (e.g., butyrate) and medium chain fatty acids (MCFA; e.g., caproate). SCFA and MCFA are absorbed into colonocytes, but MCFA absorption *in vivo* is twice as great as that of SCFA and its catabolism equals or exceeds that of SCFA [102]. Ketogenesis, an aspect of fat metabolism, is related to the host-microbiome physiology; both SCFA and MCFA are ketogenic as they are converted to the ketone body BHB in colonocytes [102, 103]. In addition to their provision of butyrate and MCFA as precursors of colonocyte BHB, gut microbes also directly generate and metabolize ketone bodies [104, 105]. Feeding of diets enriched with RS has been previously shown to increase BHB in canine feces [106] as well as in mouse plasma [107] and rat serum [90]. In this study, RS feeding significantly increased fecal concentrations of BHB. Additional substrates for production of BHB via β-oxidation, including butyrate, caproate, and 3-hydroxyhexanoate, were increased as well. As BHB inhibits neutrophil inflammasome activation [108] and activates the G-protein coupled receptor GPR109 with multitudinous metabolic benefits [109], RS might benefit colon health of cats by supporting a ketogenic colonic milieu. Co-production of butyrate alongside BHB has been proposed to generate synergistic health benefits [110]; since RS feeding increased both butyrate and BHB in the current study, these synergistic benefits might generally extend to cats consuming RS.

In addition to fatty acid catabolism, commensal gut microbes also synthesize bioactive lipid species that mimic host signaling molecules. The gut microbiome harbors diverse N-acyl amide synthases [26], and microbiota generate N-acylethanolamides [111, 112] with potential to impact host physiology [113]. Activation of the endocannabinoid pathway by N-acylethanolamides leads to loss of gut barrier integrity and metabolic endotoxemia [114], and endocannabinoids interact with the gut microbiome [115]. Feeding of prebiotic fiber has previously been reported to increase gene expression of fatty acid amide hydrolase (FAAH, degrading endocannabinoid N-acylethanolamines), decreasing arachidonoylethanolamide and restoring gut barrier integrity while decreasing circulating endotoxin [114]. In this study, we observed that RS feeding decreased a broad swath of very long chain endocannabinoid N-acylethanolamines (≥20 carbons). Since phosphatidylethanolamines were increased, RS feeding might have reduced their conversion to N-acylethanolamines. Alternatively, prebiotic RS might have increased catabolism of N-acylethanolamines by FAAH [114]. Taken together, RS feeding in the HRS group had a consistent effect to decrease very long-chain N-acylethanolamine endocannabinoids.

As in humans, cats generate primary bile acids while colonic microbiota convert these to secondary bile acids [116] with implications for host physiology. Bile acids bind the nuclear farnesoid X receptor and modulate the innate immune system to induce tolerance of the host to resident gut microbes [117, 118]. Although dysregulated production of bile acids can promote colon carcinogenic processes [119], secondary bile acids have been reported to accumulate in tumor tissue to decrease tumor growth and increase patient survival [120]. In this study, the increase of primary bile acids in the HRS-fed cats was persistent, while the effect of RS on secondary bile acids decreased over time. One of the decreases observed across the study was for the secondary bile acid deoxycholate, which activates the enzyme responsible for hydrolyzing N-acylphosphatidylethanolamines to endocannabinoid N-acylethanolamines [121]. As deoxycholate was decreased in the HRS group, consequent reduction of hydrolase activity might provide a mechanism whereby endocannabinoid N-acylethanolamines decreased while phosphatidylethanolamine phospholipids increased.

The unifying biochemical directive that may be shaping the microbial metabolism of undigested carbohydrate and protein in this study appears to be the redox balance of saccharolytic and proteolytic metabolites in equilibrium with NADH:NAD^+^ (or NADPH:NADP^+^). The hydroxyl:oxo ratio of a diverse set of microbial metabolites was significantly increased in the HRS group. Consistent with these metabolites being in equilibrium with NADH:NAD^+^, we also observed that metabolites containing the nicotinic moiety and serving as precursors to NADH synthesis were strongly increased in the HRS group. These same metabolites are used therapeutically to increase NAD(H) concentrations in vivo [56]. To our knowledge, this unified shift in NADH:NAD^+^ coupled fecal redox state has not been previously reported. It may be that the availability of ample reducing equivalents produced through microbiome catabolism of RS drives this shift of toward increased hydroxyl:oxo ratios. Monocarboxylate transporters can carry ionized redox couples into colonocytes from the luminal environment [122]. Lactate:pyruvate and associated hydroxyl:oxo redox couples may serve as two-electron shuttles, enabling communication of the redox state of the microbial milieu to the host and equilibration of NADH:NAD^+^ between commensals and colonocytes. Additionally, high concentrations of lactate and NADH shift the equilibrium away from ADP-ribosylation and its concomitant detrimental effects, including toxin activity of pathogenic bacteria [123] and inflammation [124], while promoting tissue repair [125]. Although untested in this study, based on known NAD^+^ biology, microbiota from the HRS-fed cats generating high ratios of lactate:pyruvate (accompanied by high ratios of NADH:NAD^+^) may exhibit decreased post-translational ADP-ribosylation of proteins since NAD^+^ is the source of ADP-ribose for this process [123].

There was a persistent impact of RS feeding on the fecal microbiome of cats, but RS feeding impacted a smaller percentage of bacterial genera than it did fecal metabolites. Additionally, where the bulk of changes to the metabolome were increases in the HRS-fed cats, the majority of changes to genus-level OTU were decreases. Increased fecal lactate is consistent with the microbiome data, in that a prominent lactate producer (*Lactobacillus*) was the most highly increased genus and a lactate consumer (*Megasphaera*) were among the top ten decreased genera in the HRS group at both weeks 3 and 6. Members of the phylum Acidobacteria were increased, consistent with the predilection of these bacteria for environments with lower pH as might be expected when lactate concentrations increase. Regarding proteolysis, two genera in family Peptostreptococcaceae were decreased in the HRS-fed cats (*Peptostreptococcus* and *Tepidibacter*), while *Peptococcus* in family Peptococcaceae was increased. The ratio of Bacteroidetes:Firmicutes was decreased by RS feeding in this study, while genera in the Firmicutes phylum consistently served as classifiers of RS intake. In a previous study, an increased Bacteroidetes:Firmicutes ratio in rats with CKD was seen after RS feeding [90], although the RS form used in that study was pelleted rather than extruded and thus was of a more uniform structure and homogenous molecular weight than that generated by extrusion in the current study. Those authors proposed that feeding of a uniform and homogenous RS form may have led to their observation that RS decreased microbiome diversity. In contrast, our diversity data show that several metrics of microbiome community alpha diversity, including taxa richness as well as both Shannon and Simpson indices, were increased with HRS-food feeding. Assessment of alpha diversity on a continuum indicate that increases may have been driven largely by taxa with low- to mid-range abundances rather than by differences between groups of the most abundant taxa. Taxa of intermediate abundance were the greatest contributors to differences in beta diversity.

A strength of this study is the longitudinal design whereby the persistence of the effects of dietary RS in cats can be assessed. As well, results of the preliminary digest studies, stool scoring and fecal moisture data, and food intakes show that it is feasible to implement processing modifications impacting microbiome and metabolomic features without detrimentally deteriorating stool quality, digestibility, or food acceptance. Since reduced starch cook can be associated with decreased stool firmness, it may be that in the current trial the CF and NDF levels stemming from added cellulose and beet pulp abrogated deteriorations in stool firmness that might otherwise have occurred with the relatively low percent cook of the HRS food.

A caveat to the study is that absolute concentrations of RS in the foods may have been overestimated as RS was measured in the food as ungelatinized and high molecular weight starch through enzymatic percent cook and viscosity analyses, although this should not affect the relative comparisons between intakes between groups (e.g., ratio of RS intake between HRS and LRS groups). It may, however, detrimentally impact the ability to compare to other publications that report RS per se rather than RVA or percent cook. Also, a confounding source of prebiotics in the diet was non-RS fiber. We only measured fiber in the diet as crude fiber and neutral detergent fiber, both of which may underrepresent total dietary fiber. While crude fiber was not different between the foods, neutral detergent fiber was approximately 13% decreased in the HRS food, which may have contributed in part to observed differences.

In summary, this study is the first to examine the effects of RS intake on the fecal metabolome and microbiome of cats. Cats necessarily require higher protein intakes than canine companion animals, and this predisposes them to microbial production of putrefactives with detrimental consequences to health. Provision of a grain-rich feline adult maintenance formula with a higher RS:protein ratio that reaches the colon can shift microbial metabolism toward generation of postbiotics known to benefit gut and renal health and decrease production of metabolites detrimental to health. The consumption of RS increased fecal butyrate, ketones, indolelactate/indoleproprionate, polyamines, and host production of IgA while also decreasing ammonia and endocannabinoids. As ammonia was decreased when amino acid appearance in feces was heightened, and hydroxyindoles associated with renal insufficiency were unchanged between groups, it does not appear that putrefaction to detrimental postbiotics occurred to any meaningful extent. Also, the balance of saccharolytic to proteolytic bacteria was shifted in a way that provides a mechanism to underpin the observed differences in metabolites. Cats, which are obligate carnivores, can benefit from consumption of a grain-based food that contains substantial RS.

## Supporting information

S1 Fig Extrusion under (A) LRS and (B) HRS conditions.
(TIF)

S2 Fig Sparse partial least squares analysis of whole fecal differences at weeks 3 and 6 between LRS and HRS food-fed cats for the metabolome (A,B) and microbiome (C,D). Shading indicates 95% confidence regions.
(TIF)

S3 Fig Random forest analysis of predictors of metabolomic group differences at (A) week 3 and (B) week 6.
(TIF)

S4 Fig Random forest analysis of predictors of group differences in the microbiome at (A) week 3 and (B) week 6. Operational taxonomic unit number, family, genus, and species (where known) are shown.
(TIF)

S1 Table Baseline characteristics, group assignments, and stool collections for cats in the study. (DOCX)

S2 Table Mean food intakes and undigested nutrients. (DOCX)

S3 Table Mean values at weeks 3 and 6 in fecal proximate analysis, minerals, short-chain fatty acids, selected metabolomics, IgA, and microbiome in cats fed LRS or HRS food. (XLSX)

S4 Table Loadings for sparse partial least squares analysis for the metabolome and microbiome at weeks 3 and 6. (XLSX)

S5 Table Alpha and beta diversity plots versus order q. (XLSX)
