# Supplementary figures and images for: Dietary resistant starch preserved through mild extrusion of grain alters fecal microbiome metabolism of dietary macronutrients while increasing immunoglobulin A in the cat
Source: PLoS One. 2020 Nov 3;15(11):e0241037. doi: 10.1371/journal.pone.0241037 (PMC7608938; doi:10.1371/journal.pone.0241037)

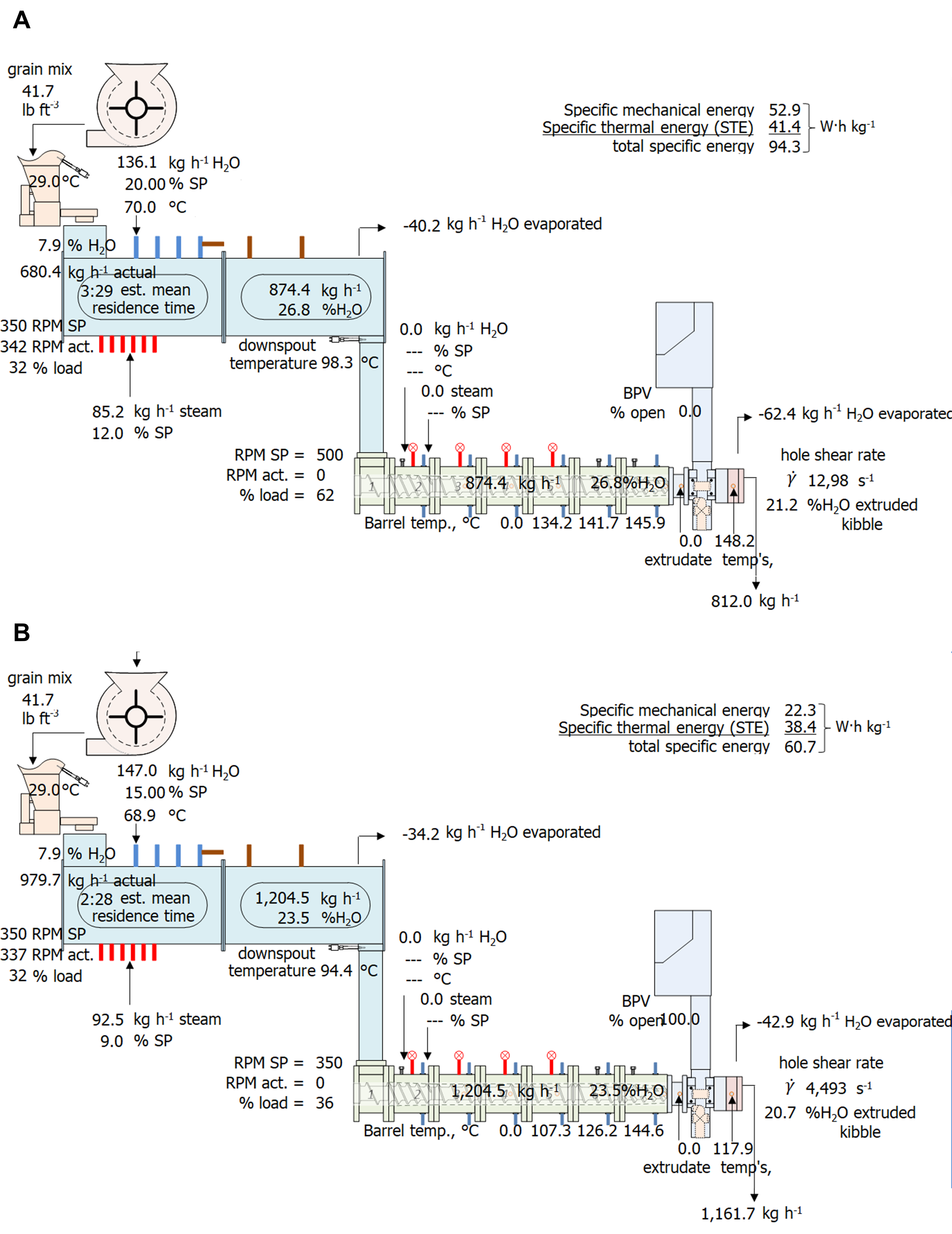

Supplement: S1 Fig — Extrusion under (A) LRS and (B) HRS conditions. (TIF) [file pone.0241037.s001.tif]

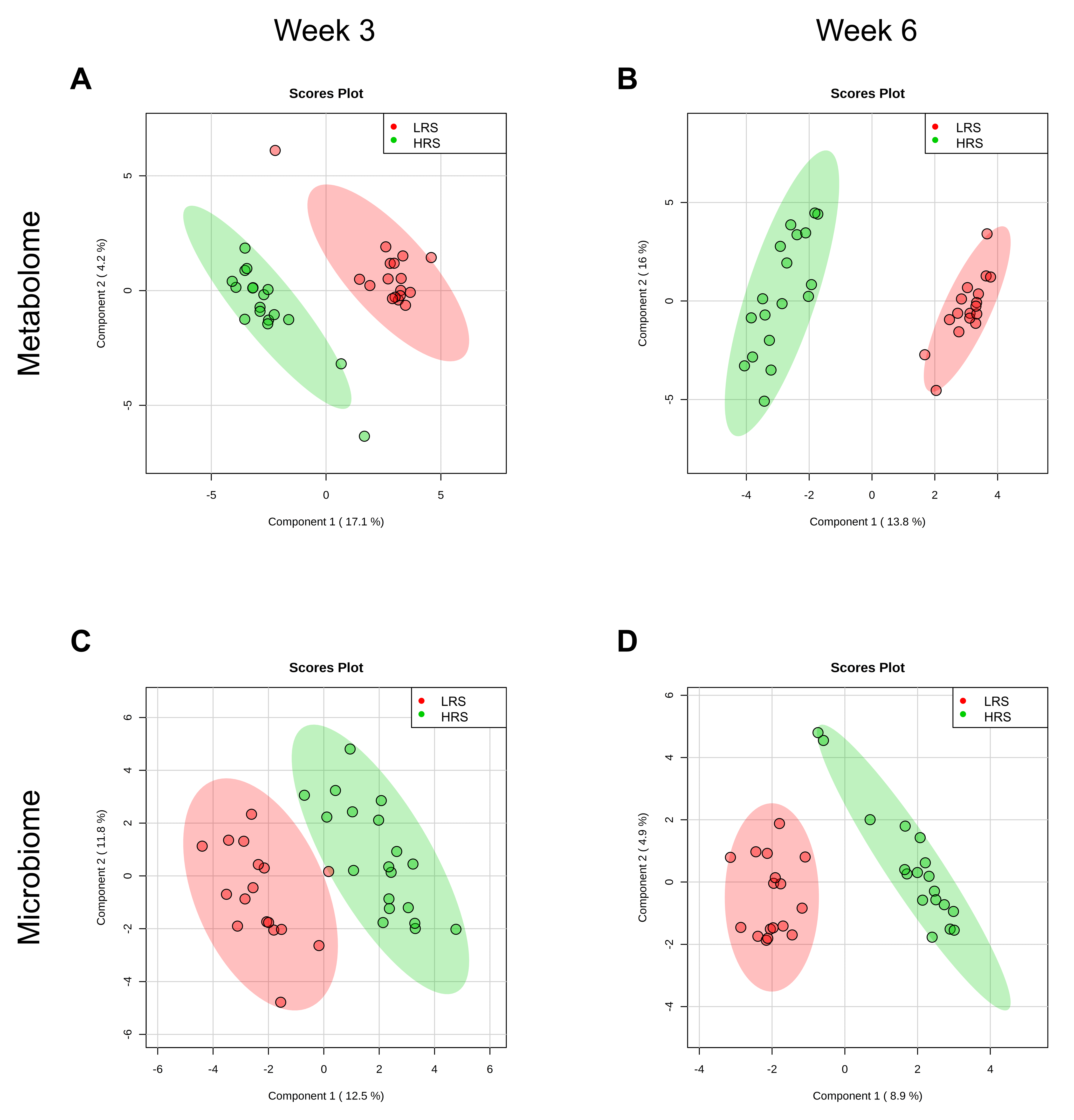

Supplement: S2 Fig — Sparse partial least squares analysis of whole fecal differences at weeks 3 and 6 between LRS and HRS food-fed cats for the metabolome (A,B) and microbiome (C,D). Shading indicates 95% confidence regions. (TIF) [file pone.0241037.s002.tif]

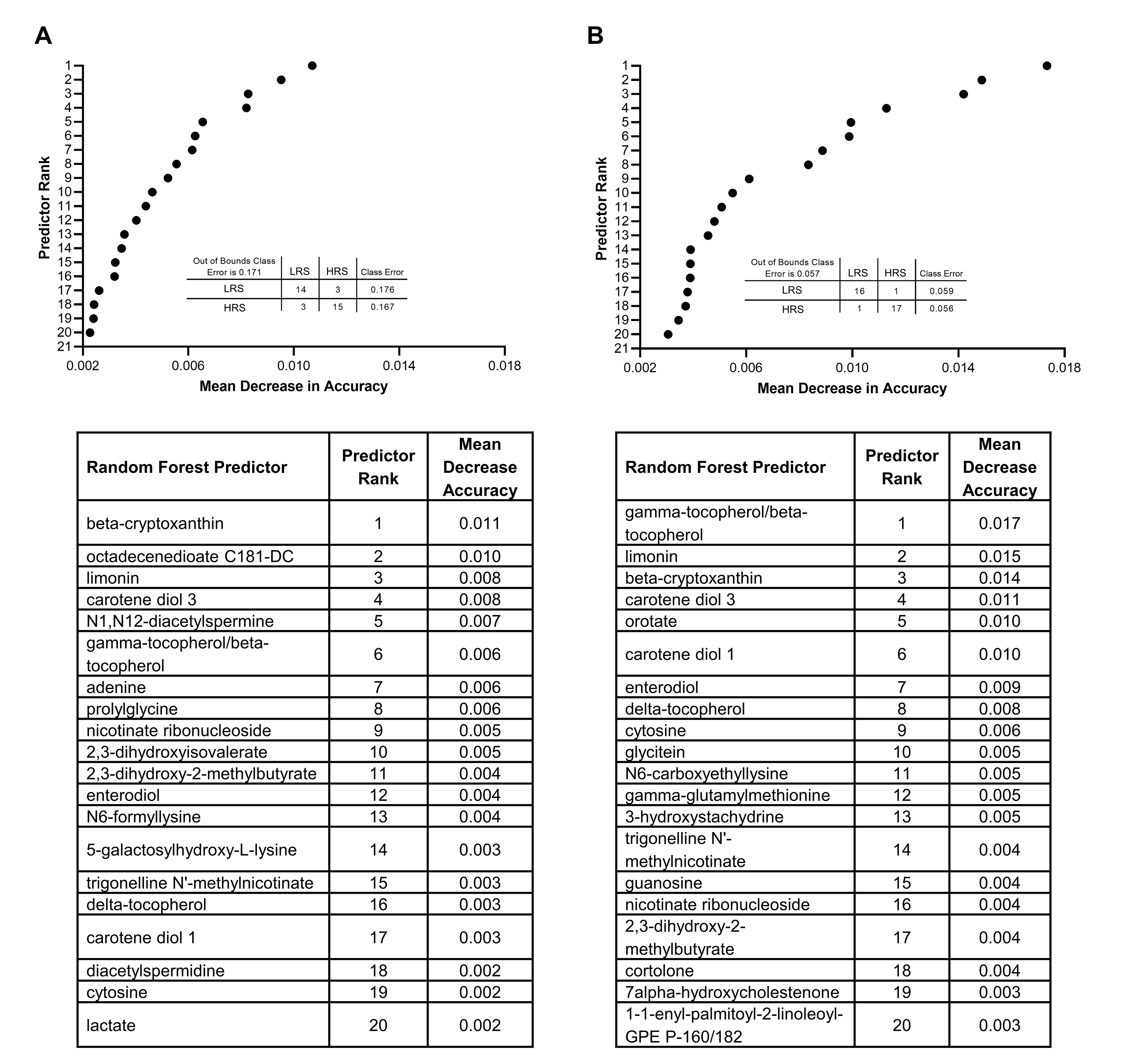

Supplement: S3 Fig — Random forest analysis of predictors of metabolomic group differences at (A) week 3 and (B) week 6. (TIF) [file pone.0241037.s003.tif]

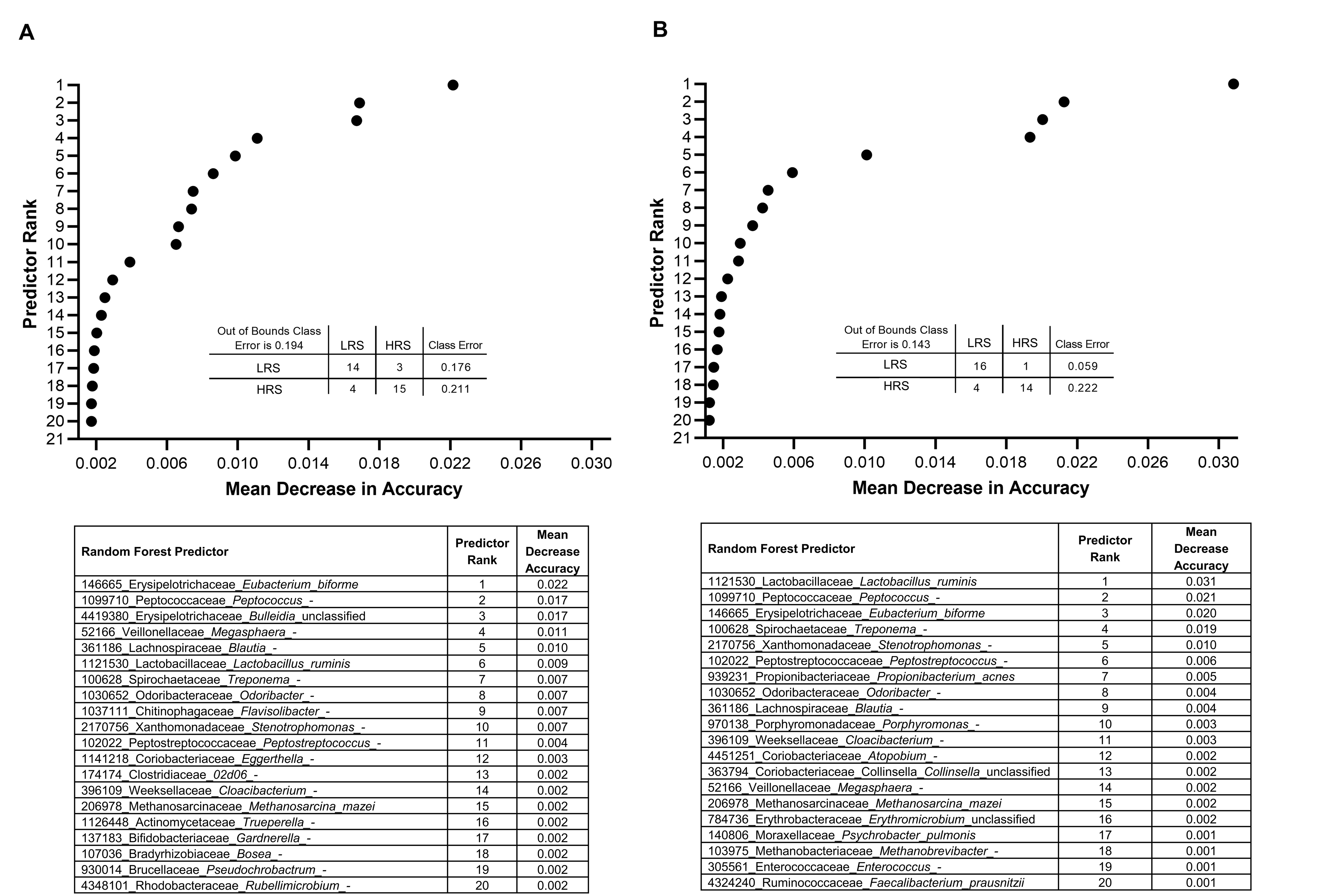

Supplement: S4 Fig — Random forest analysis of predictors of group differences in the microbiome at (A) week 3 and (B) week 6. Operational taxonomic unit number, family, genus, and species (where known) are shown. (TIF) [file pone.0241037.s004.tif]
