# Supplementary material for: Dietary resistant starch preserved through mild extrusion of grain alters fecal microbiome metabolism of dietary macronutrients while increasing immunoglobulin A in the cat
Source: PLoS One. 2020 Nov 3;15(11):e0241037. doi: 10.1371/journal.pone.0241037 (PMC7608938; doi:10.1371/journal.pone.0241037)
Supplement: S1 Table — (DOCX) [file pone.0241037.s005.docx]

**S1 Table. Baseline characteristics, group assignments, and stool collections for cats in the study.**

|  |  |  |  |  | **Week 3 feces collection** | | **Week 6 feces collection** | |
| --- | --- | --- | --- | --- | --- | --- | --- | --- |
| **Subject** | **Food group** | **Age (y)** | **Weight (kg)** | **Sex** | **First** | **Second** | **First** | **Second** |
| 1 | High RS | 10.3 | 3.39 | Spayed Female | Yes | Yes | Yes | Yes |
| 2 | High RS | 4.0 | 3.56 | Spayed Female | Yes | Yes | Yes | Yes |
| 3 | High RS | 8.2 | 4.99 | Neutered Male | Yes | Yes | Yes | Yes |
| 4 | Low RS | 3.7 | 8.27 | Neutered Male | Yes | Yes | Yes | Yes |
| 5 | Low RS | 10.1 | 4.97 | Spayed Female | Yes | Yes | Yes | Yes |
| 6 | High RS | 11.0 | 5.42 | Spayed Female | Yes | Yes | Yes | Yes |
| 7 | High RS | 10.1 | 4.05 | Spayed Female | Yes | Yes | Yes | Yes |
| 8 | Low RS | 13.5 | 3.38 | Spayed Female | Yes | Yes | Yes | Yes |
| 9 | Low RS | 11.7 | 4.54 | Spayed Female | Yes | Yes | Yes | Yes |
| 10 | High RS | 3.7 | 4.36 | Spayed Female | Yes | Yes | Yes | Yes |
| 11 | High RS | 8.4 | 5.47 | Neutered Male | Yes | No | Yes | Yes |
| 12 | Low RS | 3.9 | 5.73 | Neutered Male | Yes | No | Yes | Yes |
| 13 | Low RS | 3.8 | 5.4 | Spayed Female | Yes | Yes | Yes | Yes |
| 14 | Low RS | 10.6 | 4.51 | Spayed Female | No | Yes | Yes | Yes |
| 15 | High RS | 12.4 | 4.5 | Spayed Female | Yes | Yes | Yes | Yes |
| 16 | High RS | 10.7 | 3.78 | Spayed Female | Yes | Yes | Yes | Yes |
| 17 | High RS | 3.8 | 3.98 | Spayed Female | Yes | Yes | Yes | Yes |
| 18 | High RS | 13.4 | 3.66 | Spayed Female | Yes | Yes | No | No |
| 19 | Low RS | 8.2 | 4.59 | Spayed Female | Yes | Yes | Yes | Yes |
| 20 | High RS | 6.8 | 2.83 | Spayed Female | Yes | No | Yes | Yes |
| 21 | Low RS | 12.3 | 5.3 | Neutered Male | Yes | Yes | Yes | Yes |
| 22 | High RS | 12.3 | 3.57 | Spayed Female | Yes | Yes | Yes | No |
| 23 | Low RS | 13.1 | 3.99 | Spayed Female | Yes | Yes | Yes | Yes |
| 24 | High RS | 3.9 | 5.69 | Neutered Male | Yes | Yes | Yes | No |
| 25 | Low RS | 6.4 | 4.43 | Spayed Female | Yes | Yes | Yes | Yes |
| 26 | High RS | 6.7 | 5.8 | Neutered Male | Yes | Yes | Yes | Yes |
| 27 | Low RS | 3.8 | 3.94 | Spayed Female | Yes | Yes | Yes | Yes |
| 28 | High RS | 6.4 | 6.78 | Neutered Male | Yes | Yes | Yes | Yes |
| 29 | High RS | 8.0 | 4.99 | Neutered Male | Yes | Yes | Yes | Yes |
| 30 | Low RS | 8.2 | 5.23 | Neutered Male | No | Yes | Yes | No |
| 31 | High RS | 8.1 | 4.79 | Spayed Female | Yes | Yes | Yes | No |
| 32 | High RS | 13.2 | 4.21 | Spayed Female | Yes | Yes | No | Yes |
| 33 | Low RS | 6.7 | 6.7 | Neutered Male | Yes | No | Yes | Yes |
| 34 | Low RS | 10.9 | 5.41 | Spayed Female | Yes | Yes | Yes | Yes |
| 35 | Low RS | 13.4 | 3.29 | Spayed Female | Yes | Yes | Yes | Yes |
| 36 | Low RS | 13.5 | 3.8 | Neutered Male | Yes | Yes | Yes | Yes |
| - | Low RS | 12.3 | 4.26 | Spayed Female | No | No | No | No |
| - | Low RS | 6.4 | 6.75 | Neutered Male | No | No | No | No |
| - | Low RS | 8.1 | 4.08 | Spayed Female | No | No | No | No |
| - | High RS | 13.4 | 5.38 | Neutered Male | No | No | No | No |

Subject #18 was excluded from fecal metabolomics analysis but was included where possible for other analyses, such that for the high RS group, the week 6-only assessments for all other endpoints were n = 18 while the week 3-only assessments were n = 19. RS, resistant starch.
