# Supplementary material for: Dietary resistant starch preserved through mild extrusion of grain alters fecal microbiome metabolism of dietary macronutrients while increasing immunoglobulin A in the cat
Source: PLoS One. 2020 Nov 3;15(11):e0241037. doi: 10.1371/journal.pone.0241037 (PMC7608938; doi:10.1371/journal.pone.0241037)
Supplement: S2 Table — (DOCX) [file pone.0241037.s006.docx]

**S2 Table. Mean food intakes and bypass nutrients.**

| **Intakes** | **Mean (SE)** | | **HRS/LRS** | **t-test** |
| --- | --- | --- | --- | --- |
|  | **LRS** | **HRS** | **FC** | **p** |
| kcal | 70.6 (3.1) | 74.2 (2.2) | 1.05 | 0.191 |
| Diet, g | 16.1 (0.7) | 17.3 (0.5) | 1.07 | 0.347 |
| Nitrogen-free extract, g | 4.7 (0.2) | 4.9 (0.1) | 1.04 | 0.470 |
| Protein, g | 6.1 (0.3) | 6.6 (0.2) | 1.08 | 0.133 |
| Fat, g | 3.1 (0.1) | 3.3 (0.1) | 1.06 | 0.160 |
| RS, g | 0.1 (0.0) | 1.4 (0.0) | 18.05 | < 0.001 |
| Bypass protein, g | 0.3 (0.0) | 0.7 (0.0) | 2.08 | < 0.001 |
| Bypass fat, g | 0.2 (0.0) | 0.2 (0.0) | 1.22 | < 0.001 |

Intakes are expressed per metabolic body weight (kg^0.75^).

FC, fold change; HRS, high resistant starch; LRS, low resistant starch; SE, standard error.
